# Surgery for scapula process fractures: Good outcome in 26 patients

**DOI:** 10.3109/17453670903025394

**Published:** 2009-06-01

**Authors:** Jack Anavian, Coen A Wijdicks, Lisa K Schroder, Sandy Vang, Peter A Cole

**Affiliations:** ^1^Department of Orthopaedic Surgery, University of MinnesotaMinneapolisUSA; ^2^Division of Orthopaedic Trauma, Department of Orthopaedic Surgery, University of Minnesota, Regions HospitalSaint Paul, MinnesotaUSA

## Abstract

**Background** Generally, scapula process fractures (coracoid and acromion) have been treated nonoperatively with favorable outcome, with the exception of widely displaced fractures. Very little has been published, however, regarding the operative management of such fractures and the literature that is available involves very few patients. Our hypothesis was that operative treatment of displaced acromion and coracoid fractures is a safe and effective treatment that yields favorable surgical results.

**Methods** We reviewed 26 consecutive patients (27 fractures) treated between 1998 and 2007. Operative indications for these process fractures included either a painful nonunion, a concomitant ipsilateral operative scapula fracture, ≥ 1 cm of displacement on X-ray, or a multiple disruption of the superior shoulder suspensory complex. All patients were followed until they were asymptomatic, displayed radiographic fracture union, and had recovered full motion with no pain.

**Patients and results** 21 males and 5 females, mean age 36 (18–67) years, were included in the study. 18 patients had more than one indication for surgery. Of the 27 fractures, there were 13 acromion fractures and 14 coracoid fractures. 1 patient was treated for both a coracoid and an acromion fracture. Fracture patterns for the acromion included 6 acromion base fractures and 7 fractures distal to the base. Coracoid fracture patterns included 11 coracoid base fractures and 3 fractures distal to the base. Mean follow-up was 11 (2–42) months. All fractures united and all patients had recovered full motion with no pain at the time of final follow-up. 3 patients underwent removal of hardware due to irritation from hardware components that were too prominent. There were no other complications.

**Interpretation** While most acromion and coracoid fractures can be treated nonoperatively with satisfactory results, operative management may be indicated for displaced fractures and double lesions of the superior shoulder suspensory complex.

## Introduction

Scapula process fractures of the coracoid and acromion have been given little consideration since they have traditionally been managed nonoperatively, often with favorable outcomes. The literature regarding the operative management of these process fractures is mostly comprised of case reports and small series, and is therefore scarce and anecdotal. As a result, neither operative indications nor operative results for these process fractures are well chronicled.

Coracoid fractures most commonly occur through the base of the coracoid and are usually minimally displaced, unless there is an associated ipsilateral AC joint separation (Butters 1996). Nonoperative management is recommended for coracoid fractures with no or minimal displacement (Carr and Broughton 1989, Martin-Herrero et al. 1990, Butters 1996). Similarly, nonoperative treatment of undisplaced acromion fractures is recommended (Ada and Miller 1991, Kuhn et al. 1994, Ogawa and Naniwa 1997). These fractures often heal uneventfully with nonoperative management (McGahan et al. 1980, Armstrong and Van der Spuy 1984, Hall and Calvert 1995). Nevertheless, acromion nonunion is a widely reported complication of nonoperatively managed fractures with various different fracture patterns and varied extent of displacement (Darrach 1914, Mick and Weiland 1983, Rockwood and Matsen 1990, Kuhn et al. 1994, Douchis et al. 1999). Progressive displacement of an initially undisplaced acromion fracture has also been reported (Gorczyca et al. 2001).

We hypothesized that operative treatment of displaced coracoid and acromion fractures is a safe and effective treatment. We assessed the surgical results of open reduction and internal fixation of these fractures.

## Patients and methods

Between 1998 and 2007, all patients presenting with a highly displaced (≥ 1 cm) scapula fracture meeting criteria for operative management were enrolled in a prospective study monitoring the results of open reduction and internal fixation. All patients were managed by a single surgeon (PAC) at 1 of 2 level-1 trauma centers, which also served as a referral destination for management of scapula fractures.

Operative indications for these process fractures included a painful nonunion, ≥ 1 cm of displacement upon radiographic evaluation, a multiple disruption of the superior shoulder suspensory complex (Goss 1993), and a concomitant ipsilateral operative scapula fracture. Operative indications for these ipsilateral scapula fractures included ≥ 4 mm step-off of an articular glenoid fracture, > 20 mm of medialization of the glenohumeral joint, > 25° of angular deformity in the semicoronal plane as seen in the scapula Y view, or displaced (> 10 mm) double lesions of the superior shoulder suspensory complex (SSSC).

26 patients met the inclusion criteria for this study. Patient demographics, associated ipsilateral upper extremity injuries, fracture classification/pattern, method of fixation, surgical results, and postoperative complications were recorded. Fractures were classified according to the AO/OTA classification (Kuhn et al. 1994, Eyres et al. 1995, Ogawa et al. 1996, Ogawa and Naniwa 1997, Marsh et al. 2007). Radiographic studies, including anteroposterior (AP), scapula Y (lateral), and axillary radiographs were done for all patients. In addition to standard shoulder radiographs, 2D-CT and 3D-CT reconstructions were obtained for patients with concomitant glenoid or scapular neck/body fractures.

Views that best visualized the fracture line in the plane represented by the standard scapula were collected for each patient and imported into Macromedia Fireworks MX software to overlap and orient fracture patterns onto a template scapula image. Images of each scapula were graphically superimposed to create a compilation of fracture lines on a standard scapula serving as the foundation for the bony anatomy of all patient fracture maps. The overlap of all major fracture lines resulted in a frequency diagram based on the density of fracture lines. Once proper anatomical alignment was obtained, fracture lines were identified and were traced on top of the combined 3D-CT and model scapula. Fracture patterns were then confirmed using the original 3D-CT rendering.

### Surgical technique for coracoid fractures

Most coracoid fractures are fixed through a direct incision over the coracoid in Langer's lines. This may be a proximal extension of the deltopectoral approach, if a concomitant anterior or superior glenoid fracture is being addressed. The surgeon must dissect down the cephalad slope of the coracoid to its base until the fracture is appreciated and can be reduced anatomically, mobilizing the coracoclavicular ligaments for proper visualization. A simple 3.5-mm lag screw is often all that is needed for adequate stability. A one-quarter tubular plate along the cephalad slope spanning the fracture may also be warranted. In cases where a coracoid base fracture involves a portion of the superior glenoid fossa (type V according to Eyres et al. 1995), fixation of the coracoid can be achieved indirectly through a posterior approach, and then indirect reduction of the superior glenoid with attached coracoid is accomplished from behind.With this scenario, typically 2.7-mm reconstruction plates are used with cortical lag screws through the acromial spine to fix the superior glenoid fragment, which is attached to the spinoglenoid notch and coracoid.

### Surgical technique for acromion fractures

All acromion fractures in this study were treated using a posterior approach. An incision is made over the posterior acromion border and between the fascia of the deltoid and trapezius muscles. The deltoid is elevated off the posterior aspect of the acromion spine and reflected with the infraspinatus. This will allow for appreciation of the neck, the base, and the entire acromial spine. Transverse fractures can be directly clamped perpendicular to the fracture line with a small, pointed bone tenaculum. Strategic drill holes help to notch the bone on either side of the fracture, to allow the tenaculum to be captured for optimal fracture compression. In addition, one or two 2.7- or 3.5-mm lag screws may be applied perpendicular to the fracture for compression. Lag screw holes should be countersunk in superficial areas to prevent any prominence of hardware. If the fracture is through the neck or base of the acromion, a 2.4- or 2.7-mm reconstruction plate should be used in a neutralization mode to distribute stress for optimum stability. If the fracture involves the spine of the scapula, just proximal to the base, a 3.5-mm lag screw can be inserted into the glenoid neck.

For distal acromion fractures, conventional plate fixation may not be optimal due to the thin bone. A tension band technique should be considered to permit rotational control of the distal fragment and compression at the fracture site. Alternatively, the fracture may be fixed with a thin locking plate over the superior surface, which can be achieved with short locking screws, or with a plate placed on the anterior or posterior acromial edge. If placed along the anterior or posterior edge, the surgeon must make sure to negotiate screws into the thin acromion and not into the subacromial space.

### Postoperative management and follow-up

Postoperatively, all patients were placed in a sling for comfort and physiotherapy was started immediately. The therapy protocol began with passive and active assisted range-of-motion exercises for a period of 1 month followed by active range-of-motion exercises with no restrictions in motion for 1 month. Patients were prevented from carrying heavy objects during this time. Resistance and gradual strengthening exercises, starting with 3–5 pound weights was started after 2 months. By this time, there was early radiographic evidence of healing. All restrictions were removed by 3 months.

All patients from both institutions were followed at least until they had full range of motion, were not tender at the fracture site, and displayed evidence of healing—which was defined as fracture union seen on radiographic evaluation at ≥ 2 months following surgery. Follow-up radiographs included AP, scapula Y, and axillary views of the shoulder; they were reviewed by 3 observers.

Quantitative functional results including shoulder range of motion, shoulder strength, evidence of residual pain, and return to prior occupation and activities were obtained for patients treated at the current institution of the senior author (PAC). Shoulder motion was measured in degrees using a goniometer and shoulder strength was measured in pounds of force using a hand-held dynamometer. The Disabilities of the Arm, Shoulder and Hand (DASH) questionnaire (Hudak et al. 1996) and Short Form-36 (SF-36) version 2 (Ware et al. 1994) were also used to assess functional outcome in these patients. All complications were investigated and reported.

### Ethics

The procedures followed in this study were in accordance with the ethical standards of the responsible committee on human experimentation, and with the Helsinki Declaration of 1975, as revised in 2000. The study design and enrollment process complied with all guidelines of the respective Institutional Review Boards (date of issue: August 5, 2003; reference no. 02-087).

## Results

Of 156 patients with scapula fractures, 26 patients (27 closed fractures, 21 males; mean age 36 (18–67) years) underwent operative management for a coracoid and/or acromion fracture (Table 1 of Supplementary data). Of the 27 fractures, there were 13 acromion fractures (OTA type A1.1) and 14 coracoid fractures (OTA type A2.1). 1 patient (no. 5) was treated for both a coracoid fracture and an acromion fracture. Fracture patterns for the acromion included 6 acromial base fractures, 4 proximal acromion fractures, and 3 fractures midway along the acromion. Coracoid fracture patterns included 11 coracoid base fractures and 3 fractures midway along the coracoid. (Table 2, see Supplementary data).

**Figure 1.**
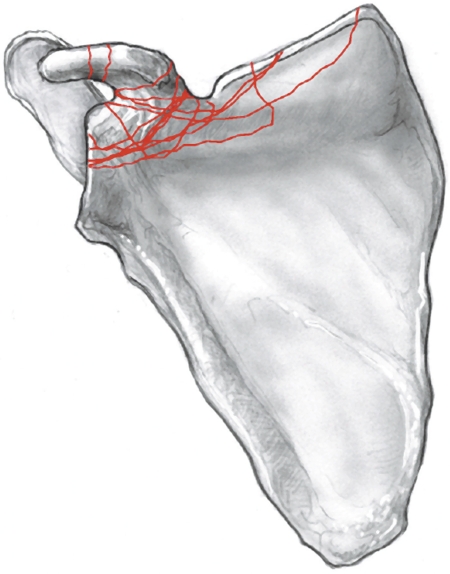
AP illustration of the scapula showing the 14 coracoid fracture patterns seen in this cohort. The patterns together yield the “coracoid fracture map.”

**Figure 2.**
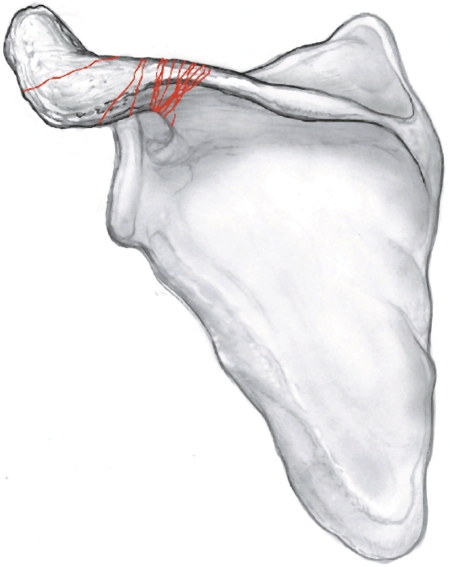
AP illustration of the scapula showing the 13 acromion fracture patterns seen in this cohort. The patterns together yield the “acromion fracture map.”

Overlay of the fractures yielded illustrations of the coracoid and acromion fracture patterns and demonstrated the morphological variation with which these fracture patterns occurred (Figures 1 and 2). The overlaid fracture patterns followed a reproducible, non-random path. This observation was more apparent for acromion fractures, in that there was a recurring pattern at the base of the acromion.

**Figure 3:**
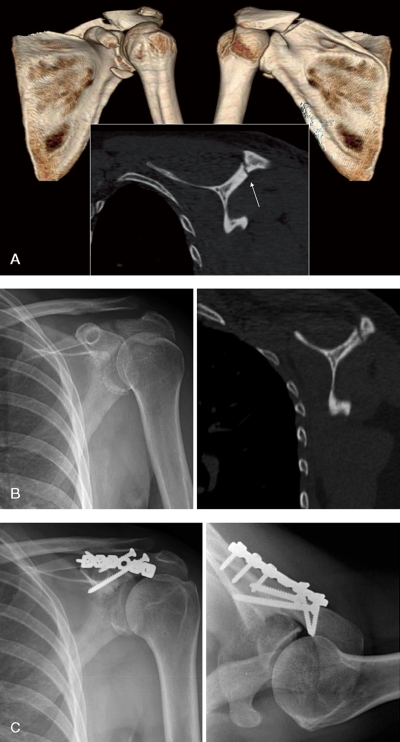
A. 2D- and 3D-CT scan of the scapula (patient 9) showing a minimally displaced fracture of the acromial base at the time of injury. B. AP radiography (left) and 2D-CT (right) of the scapula in the same patient 4 months after the injury, showing a nonunion of the acromial base. The patient complained of tenderness over the acromion and significant pain on movement of the shoulder. C. AP (left) and axillary (right) radiographs postoperatively, illustrating fixation using a 6-hole reconstruction plate, contoured to the acromial base, supplemented with two 3.5-mm cortical lag screws.

**Figure 4:**
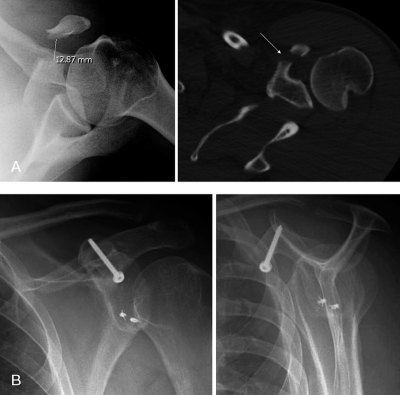
A. AP radiograph (left) and CT scan of the scapula (right) showing fracture of the middle coracoid with > 1 cm of displacement. B. AP (left) and lateral (right) radiographs after open reduction and internal fixation using a cortical lag screw.

Operative indications included a painful acromion base nonunion in 2 patients (Figure 3), a concomitant ipsilateral operative scapula fracture in 16 patients, ≥ 1 cm of fracture displacement in 16 patients (17 fractures) (Figure 4), and a multiple disruption of the superior shoulder suspensory complex in 16 patients. 18 patients had more than one criterion for surgery. (Table 1, see Supplementary data).

The method of fixation for coracoid fractures included cortical lag screw placement in all 14 cases (Figure 4) with supplemental plate fixation in 7 of these cases. The method of fixation for acromion fractures included plate fixation in 4 cases and plate fixation with supplementary cortical lag screws in 9 cases (Table 2 of Supplementary data). Average follow-up time was 11 (2–42) months. All fractures had united at follow-up. All patients had recovered full motion without pain at the time of final follow-up.

8 of the 26 patients were treated at the previous institution of the senior author (PAC), at which time quantitative functional outcomes were not formally obtained as part of follow-up visits. Of the remaining 18 patients that were treated at the senior author's current institution, functional outcomes were obtained for 13 patients at a mean follow-up of 22 (12–42) months (Table 3, see Supplementary data).

All 13 patients were pain-free at rest and when using the affected upper extremity. The mean DASH score was 7 (0–26) for this subset of patients, compared to the mean normative DASH score for the uninjured population of 10. The mean SF-36 scores in all 8 parameters for the study patients were similar to the mean scores of the normal population. (Study mean scores across all parameters were 68–97 points, compared to control mean scores of 61–84 points). A lower DASH score on a 100-point scale translates into a better outcome, and a higher SF-36 score on a 100-point scale also reflects a better outcome. Patient 14 sustained a T12 spinal cord paralysis as a result of his injury; therefore, DASH and SF-36 scores could not be obtained for this patient. Patient 17 sustained a severe brachial plexus injury such that he had no use of the injured upper extremity. Even so, because he had complete use of his contralateral, uninjured extremity, a DASH score and SF-36 scores were obtainable although range-of-motion and strength measurements could not be obtained. All of these patients, with the exception of patients 14 and 17, returned to their previous occupations and activities.

Patient 8 underwent complete removal of hardware due to prominence and irritation over the acromion. Another patient (no. 19) experienced hardware loosening, whereby a screw had backed out from a reconstruction plate applied to the acromion, causing irritation to the surrounding tissue. This screw was removed operatively. Patient 20, treated for a coracoid fracture, required excision of ectopic bone impinging on the conjoint tendon. There were no wound complications, infections, or malunions in this series.

## Discussion

Scapula fractures are uncommon; they account for 1% of all fractures (Imatani 1975, Thompson et al. 1985). Of these, only 2–13% involve the coracoid process (Wilber and Evans 1977, Ada and Miller 1991) and 8–10% involved the acromion (Wilber and Evans 1977, McGahan et al. 1980). Coracoid fractures can occur as a result of direct trauma (Froimson 1978), indirect trauma secondary to dislocation of the humeral head (Bencherit and Friedman 1979), axial loading of an associated ipsilateral clavicle fracture (Martin-Herrero et al. 1990), or avulsion by the coracoclavicular ligaments during acromioclavicular (AC) joint separation (Montgomery and Loyd 1977, Ishizuki et al. 1981) or by the muscular attachments (Rounds 1949). Reported mechanisms of injury for acromion fractures include direct trauma, indirect trauma due to dislocation of the humeral head (Goss 1996), avulsion by the deltoid muscle (Butters 1996), and stress fracture in athletes and patients who have undergone subacromial decompression (Marr and Misamore 1992, Warner and Port 1994). These are predominantly due to high-energy/high-velocity mechanisms of injury, the most common being motor vehicle accidents (Imatani 1975, McGahan et al. 1980).

Under most circumstances, undisplaced or minimally displaced coracoid or acromion fractures can be managed nonoperatively with satisfactory results (Carr and Broughton 1989, Martin-Herrero et al. 1990, Ada and Miller 1991, Kuhn et al. 1994, Butters 1996, Ogawa and Naniwa 1997). Even so, complications of nonoperative management of these process fractures have been reported. Symptomatic nonunion is a reported complication of both coracoid and acromion fractures (Darrach 1914, Garcia-Elias and Salo 1985, Douchis et al. 1999). Goss (1993) reported that a fracture of the coracoid and/or acromion that occurs as part of a double disruption of the superior shoulder suspensory complex can result in an unstable anatomical situation that can ultimately result in adverse healing and functional outcomes. Neer (1984) described compression of the brachial plexus and also paralysis of the suprascapular nerve in patients with coracoid base fractures, and recommended diagnostic electromyography followed by early exploration. Nonoperative treatment of coracoid fractures in athletes and patients involved in heavy manual labor may result in poor outcome (Guttentag and Rechtine 1988, Goss 1996). With regard to nonoperatively managed acromion fractures, a wide variety of long-term complications have been reported, including pain, reduced motion, rotator cuff tears secondary to subacromial impingement, AC joint separation, humeral head subluxation, shoulder weakness, and brachial plexus injury (Wilber and Evans 1977, Hardegger et al. 1984, Rockwood and Matsen 1990, Kuhn et al. 1994, Gorczyca et al. 2001).

The indications for operative management and its safety and efficacy in the treatment of these fractures are yet to be established. To date, the largest operative series for coracoid and acromion fractures include 35 patients (of 67 with fracture) and 8 patients (of 37 with fracture), respectively, both described by Ogawa et al. (1996) and Ogawa and Naniwa (1997). We used 4 criteria for operative treatment of coracoid and acromion fractures, including symptomatic nonunion, concomitant ipsilateral scapula fracture, ≥ 1 cm of displacement upon radiographic evaluation, and/or a multiple disruption of the superior shoulder suspensory complex (SSSC). The SSSC is the bony and soft tissue ring of the shoulder girdle that suspends the upper extremity from the thorax. This ring is composed of the glenoid process, acromion, acromioclavicular ligament, clavicle, coracoclavicular ligaments, and the coracoid process. An isolated disruption of this ring is generally well tolerated; however, multiple disruptions of this ring will often create an unstable anatomic situation, which may result in adverse healing and functional outcomes (Goss 1993, Rikli et al. 1995). In addition to the 4 operative criteria used in this series, various operative indications have been alluded to in the literature, many of which would apply to our patients.

Operative techniques described for open reduction and internal fixation of coracoid fractures include interfragment screw fixation with cortical lag screws for larger fragments (Eyres et al. 1995, Goss 1996, Ogawa et al. 1996) and nonabsorbable suture for small fragments (Butters 1996). In our series, all 14 coracoid fractures were fixed with cortical lag screws. In 7 of these fractures that involved the superior glenoid fossa (Ideberg type III; n = 4) or the superior border of the scapula body (n = 3), fixation was supplemented with a 1/4- to 1/3-tubular or reconstruction plate.

A variety of fixation techniques have been described for the open reduction and internal fixation of acromion fractures, including tension band wiring for more distal fractures (Butters 1996, Goss 1996, Lim et al. 1996, Ogawa and Naniwa 1997), plate fixation for fractures that are more proximal or through the acromial base and spine (Butters 1996, Goss 1996, Ogawa and Naniwa 1997), interfragment screw fixation (Mick and Weiland 1983), plate fixation supplemented with interfragment screws (Douchis et al. 1999), and fixation with Kirschner wires (O'Donoghue 1960, Ogawa and Naniwa 1997). In our series, techniques for the fixation of the acromion included plate fixation in 4 cases and plate fixation with supplementary cortical lag screws in 9 cases.

In this article we have reported the clinical and surgical results of 26 operatively managed acromion or coracoid fractures. All of the patients in this series were followed until they had pain-free range of motion, had no tenderness at the fracture site, and displayed fracture union upon radiographic evaluation. Quantitative functional outcomes were obtained for 13 of these patients, which showed a good to excellent overall outcome. The mean DASH score was 7, which is lower than the normative mean score for an uninjured population (10). The mean SF-36 scores in all 8 parameters were comparable to the normative mean scores, and in some parameters these patients scored higher than the normative mean. Soft tissue irritation due to prominent hardware is a minor reported complication (Douchis et al. 1999) that occurred in 2 of our patients and was treated by removal of hardware, which was followed by an uncomplicated postoperative recovery. One patient required removal of ectopic bone. There were no other complications in this series.

A limitation of our study is that our cohort was limited to operatively managed process fractures; thus, we are unable to compare treatment results in nonoperative patients. Our study was not designed to be able to answer the question of whether patients will do better with nonoperative or operative management. We cannot therefore state definitively when to or when not to manage a coracoid or acromion fracture operatively. Our intention with this study was to elucidate operative complications and rate of healing of the fracture, and also to describe fracture patterns and the fixation strategy employed. Our study only demonstrates that in experienced hands operating on a coracoid or acromion fracture with a certain indication, displacement of > 1 cm, is safe and can result in successful fracture union and pain-free motion.
